# Hematologic follow-up of metamizole-associated Heinz body anemia in dogs

**DOI:** 10.3389/fvets.2026.1913770

**Published:** 2026-09-28

**Authors:** Cathrina Richter, Vera Geisen, Jelena Palić, René Dörfelt

**Affiliations:** 1 LMU Small Animal Clinic, Centre for Clinical Veterinary Medicine, Ludwig-Maximilians-Universität in Munich, Munich, Germany; 2 Division of IDEXX Laboratories, Vet Med Labor GmbH, Kornwestheim, Germany

**Keywords:** dipyrone, drug-associated hematotoxicity, eccentrocytes, erythrocyte, hemolysis, oxidative stress

## Abstract

**Introduction:**

Heinz body (HB) anemia is uncommon in dogs. Metamizole is described as a potential trigger in isolated cases.

**Aim of the study:**

The present study describes the course of metamizole-associated HB anemia in dogs with the development of hematocrit, HBs, and eccentrocytes. In addition, the correlation between HBs, eccentrocytes, hematocrit, and reticulocytes is investigated.

**Materials and methods:**

In this prospective observational study, 25 dogs with metamizole-associated HB anemia were included. Hematologic examinations and blood smear evaluations for quantification of HBs and eccentrocytes (stained with brilliant cresyl blue and modified Wright's stain) were performed on day 0 and at the control intervals 1–3, 4–6, 7–13, and 14–20 days after discontinuation of metamizole and initiation of antioxidant treatment. Known alternative causes of oxidative erythrocyte damage were excluded. Data were analyzed using Kruskal-Wallis test, Friedman test, and Spearman correlation.

**Results:**

HB anemia was detected a median of 10 days after initiation of metamizole (median cumulative dose 720 mg/kg). On day 0, HB percentage did not correlate with metamizole dose or hematocrit. The eccentrocyte count did not correlate with metamizole dose, hematocrit, or reticulocyte count. However, a moderate to strong correlation was identified between HB percentage and reticulocyte count (*r* = 0.630). Median HBs and eccentrocytes decreased from 26.5% and 2.6% on day 0 to 1.3% and 0% on days 14–20, while median hematocrit increased from 24 to 33%. Four dogs received blood transfusions. All patients had at least one concurrent disease. Twenty-one dogs survived and four dogs were euthanized or died.

**Conclusion:**

In dogs with concurrent disease, metamizole administration may be associated with clinically relevant erythrocyte oxidative damage. Discontinuation of metamizole and initiation of antioxidant therapy were followed by a decrease in HBs and recovery of hematocrit. The use of metamizole in multimorbid dogs should be carefully considered and accompanied by regular hematology controls.

## 1 Introduction

Heinz bodies (HBs) are formed in the erythrocytes due to oxidative stress on hemoglobin. After oxidation, hemoglobin is denatured and accumulates on the inner membrane surface of the erythrocyte, forming HBs, which are considered pathological in dogs (1, 2). HB containing erythrocytes become more rigid and are therefore unable to pass normally through the splenic sinusoids, leading to their mechanical destruction or phagocytosis by splenic macrophages, causing mild to severe anemia (3). Documented causes of HB anemia in dogs are intoxications with benzocaine (4, 5), zinc (6, 7), methylene blue (5, 8), acetaminophen (9, 10), phenylhydrazine (11), naphthalene (12), vitamin K1 and 3 (13), skunk musk (14, 15), propofol (16), the ingestion of allium plants (17–20) or potentially due to fragrance products (21).

Metamizole is a non-opioid analgesic which is used as a standard analgesic treatment for dogs in some regions of the world (22). In a recent retrospective case series, metamizole administration was associated with HB formation and hemolytic anemia in dogs (23).

Few studies describe oxidative damage to erythrocytes after the administration of metamizole at recommended doses. A prospective study in dogs demonstrated metamizole induced oxidative damage and eccentrocyte formation, but no HB formation (24). In a retrospective case series HB formation was described in 13 dogs with multiple comorbidities following metamizole administration. The case series study evaluated the correlation between hematocrit (HCT) and the number of HBs, but no significant correlation was found (23). The course of the metamizole-associated HB anemia in dogs is currently poorly characterized.

The aim of this study was to evaluate the course of metamizole-associated HB anemia, HCT, HBs and eccentrocytes and the correlations of HBs and eccentrocytes with HCT, reticulocytes and metamizole dose in dogs, until the hematocrit returned to the reference range or HBs were no longer detectable.

## 2 Materials and methods

This prospective, observational study was conducted over 4 years at the LMU Small Animal Clinic, Centre for Clinical Veterinary Medicine, Ludwig-Maximilians-Universität in Munich, Germany. The study was approved by the Ethics Committee of the Center for Clinical Veterinary Medicine, LMU Munich, Germany (370-18-08-2023).

### 2.1 Animals

The study included dogs with HCT values below the reference range and a HB concentration exceeding 5% in patients receiving metamizole within 1 week before the detection of anemia. The amount of HB was asumed to be significant over 5% according to the current literature (25). Dogs that received or ingested substances, known to cause HB anemia, such as allium plants, zinc, or acetaminophen, were excluded. Dogs were hospitalized or managed ambulatory depending on the severity of anemia and the presence of comorbidities. A total of 25 dogs fulfilled the inclusion criteria.

In addition to symptomatic therapy and discontinuation of metamizole therapy, all patients received antioxidative treatment with S-Adenosylmethionine (SAMe) or N-acetylcysteine (NAC). SAMe was administered at a loading dose of 40 mg/kg per os (PO) twice daily for 3 days, followed by 20 mg/kg PO twice daily until HBs were undetectable. NAC, administered at a dose of 70 mg/kg intravenously (IV) every 6 h for two consecutive days. The antioxidant therapy was chosen at the discretion of the primary veterinarian. Dogs receiving a blood transfusion during the follow-up period were excluded from analysis of the red blood cell related parameters [HCT, hemoglobin, red blood cells (RBC)] from the time of transfusion. The occurrence of nausea, vomiting, anorexia, or restlessness immediately following SAMe administration was defined as a criterion for discontinuation.

### 2.2 Data collection

A detailed medical history, including comorbidities was obtained from each patient. For metamizole, the indication, route of application, duration, dosing interval, and dosage were determined. Furthermore, signalment, physical examination findings, laboratory results, diagnostic imaging, diagnoses, comorbidities, and outcome were documented. As many dogs were treated ambulantly, the checkups were determined according to the patients' needs and grouped into intervals as listed below. Hematological results were already available for some patients prior to day 0. These results were also included in the study and comprise control intervals **–**2 and **–**1 (Table 1). To ensure a reliable analysis, prospective values were evaluated from patients with results from at least two consecutive checkups starting at day 0 to checkup 4 using repeated measures analysis. Checkups were continued until either HBs disappeared or the HCT returned to the reference range.

**Table 1:** Checkup intervals for 25 dogs included in the study with metamizole associated Heinz body anemia.

Checkup No.:	Time interval from detection of HB anemia
−2	−10 to −4 days
−1	−3 to −1 days
0	first day of HB detection
1	1–3 days
2	4–6 days
3	7–13 days
4	14–20 days

No.: Number.

At day 0 a complete blood count (CBC), including reticulocyte count was performed on in-house IDEXX ProCyte Dx Hematology Analyser. Serum chemistry including bilirubin, total protein, and albumin (performed on Cobas Integra 400 Plus; Roche Diagnostics GmbH, Switzerland), manual HB and eccentrocyte counts, and an abdominal ultrasound were carried out to detect causes for gastrointestinal bleeding or causes for hemolysis. Subsequent checkups involved a CBC, with HB and eccentrocyte quantification. Brilliant cresyl blue stained blood smears were used for the detection of HBs. Blood smears stained with modified Wright's stain were examined to detect the presence of eccentrocytes. The percentage of HBs and eccentrocytes was determined by counting 500 erythrocytes per blood smear (26). HB and eccentrocyte numbers were evaluated by a board-certified veterinary clinical pathologist (Diplomate, American College of Veterinary Pathologists, Clinical Pathology).

### 2.3 Statistical analysis

Statistical analysis was performed using commercially available software (Prism 5 for Windows; GraphPad Software, San Diego, USA). Normality was analyzed using the D'Agostino and Pearson Omnibus normality test, non-normal data are presented as median and range (minimum—maximum). Differences in values between checkup timepoints −2 and 4 were evaluated using the Kruskal Wallis test with *post hoc* Dunn's multiple comparison test. Between day 0 and other checkups analyzing only patients with full datasets, the Friedman test with *post hoc* Dunn's multiple comparison test was used. Correlation was analyzed with the Spearman correlation test. A *p*-value < 0.05 was considered statistically significant. The graphics and illustration were created using matplotlib v3.7.2 in python v3.11 and Inkscape v1.4, respectively.

## 3 Results

A total of 25 dogs were included in the study. The median age was 9 years (1–14 years), 14/25 were female (11/14 neutered) and 11/25 male (9/11 neutered). The median weight was 15.0 kg (2.5–47.8 kg). Breeds included were mixed breed dogs (4), Labrador Retriever (5) and one of each of the following breeds: Pomeranian, Miniature Pinscher, Dalmatian, Pug, Norfolk Terrier, Beagle, Rhodesian Ridgeback, Pekingese, Siberian Husky, Maltese, German Hunting Terrier, Chihuahua, Russkaya Tsvetnaya Bolonka, Labradoodle, Border Collie and Nederlandse Kooikerhondje.

All dogs received metamizole at a median dose of 105 mg/kg/d (50–155 mg/kg/d), administered 1–3 times daily via IV, oral or subcutaneous (SC) route. The median duration of treatment before detection of HBs was 10 days (3–19 days), and the median cumulative metamizole dose until detection of HBs was 720 mg/kg (290–1,656 mg/kg**)**. Additional data regarding the median and range of metamizole dosage, duration, and hospitalization are available in the Supplementary Table 1.

Not all study patients participated in every checkup and were excluded temporarily or permanently. Due to clinical signs of anemia four dogs (patient 1, 4, 12, 16) received blood transfusions. HCT-, RBC- and hemoglobin (HBG)-values were excluded from analysis from the time of transfusion. In 11 dogs (patient 3, 7, 9, 10, 14, 15, 17, 20, 23, 24, 25) subsequent or interim controls were performed by the referring veterinarian or declined by the owner and were therefore not available for the study. Overall 21 dogs received inpatient treatment, of which three (patient 2, 13, 21) were euthanized and one (patient 11) died. The reasons for euthanasia in these three patients were multimorbidity, progressive deterioration of general health and laboratory parameters, including HCT, despite intensive therapy. The anemia was caused by more than one factor, so it could not be attributed only on the presence of HBs. Patient No. 11 had mitral valve endocardiosis (Chief C) and was admitted for weakness and dyspnea secondary to pulmonary edema. Despite intensive treatment, the patient died on the second day of hospitalization (Figure 1).

**Figure 1:**
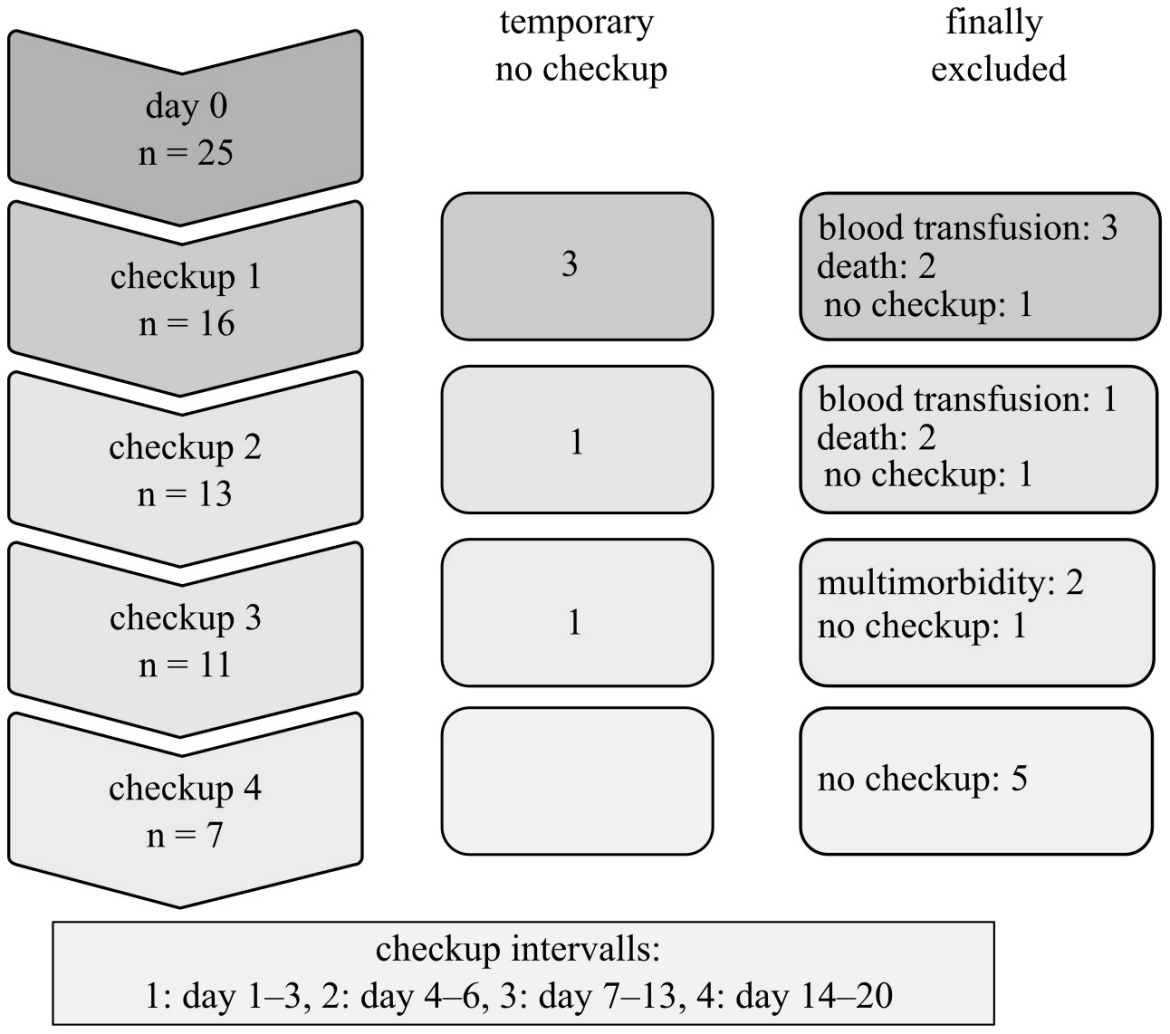
Overview of number (n) of patients included at the different checkups and causes of exclusion from the study or single checkups (Inkscape).

For nine dogs and 14 dogs hematology values were available 4–10 days (checkup −2) and 1–3 days (checkup −1) before detection of HB anemia. Analyzing all available values from checkup −2 to checkup 4 HCT, HGB and RBCs decreased from checkup −1 to day 0 and checkup 1 and increased thereafter (Table 2). The number of patients with increased, within-reference-range, or decreased values for each individual blood parameter is provided in the Supplementary Table 2.

**Table 2:** Complete blood count and selected laboratory parameters of study participants with metamizole-associated Heinz body anemia before, at, and after detection of the Heinz bodies.

Value	Checkup −2	Checkup −1	Checkup 0	Checkup 1	Checkup 2	Checkup 3	Checkup 4	*p*-value
RBC (5.65–8.87 10^12^/l)	6.1 (3.5–8.8) *n* = 9	3.5 (2.0–6.1)^*^n = 14	3.3 (1.9–5.0)^*^n = 25	3.2 (2.4–5.4)^*^n = 16	3.9 (2.4–4.6)^*^n = 13	4.7 (3.4–5.7) *n* = 11	5.0 (3.5–6.2)^#, t^ *n* = 7	**< 0.001**
HCT (37.3–61.7 l/l)	0.38 (0.26–0.57) *n* = 9	0.24 (0.14–0.43)^*^n = 14	0.24 (0.14–0.35)^*^n = 25	0.24 (0.17–0.38)^*^n = 16	0.27 (0.20–0.33) *n* = 13	0.35 (0.22–0.42)^#, t^ *n* = 11	0.33 (0.23–0.45) *n* = 7	**< 0.001**
HGB (13.1–20.5 mmol/l)	8.3 (5.2–13.2) *n* = 9	4.8 (3.2–9.3)^*^n = 14	4.6 (2.9–7.7)^*^n = 25	4.9 (3.3–8.1)^*^n = 16	5.7 (4.0–7.0) *n* = 13	7.1 (4.9–8.7)^#^ *n* = 11	6.9 (5.0–9.4) *n* = 7	**< 0.001**
MCV (61.6–73.5 fl)	63.2 (59.0–72.9) *n* = 9	70.5 (56.5–86.8) *n* = 14	69.7 (61.1–87.1) *n* = 25	72.5 (62.8–88.2)^*^n = 19	76.1 (64.9–97.1)^*^n = 16	73.1 (65.0–89.7)^*^n = 13	67.1 (61.2–75.1) *n* = 7	**0.004**
MCH (21.2–25.9 pg)	22.5 (20.8–25.8) *n* = 9	24.1 (17.1–25.8) *n* = 14	23.0 (17.4–33.8) *n* = 25	24.1 (21.3–25.6) *n* = 19	24.4 (21.6–26.7) *n* = 16	23.5 (21.3–27.6) *n* = 13	22.9 (20.9–26.4) *n* = 7	0.253
MCHC (32–37.9 g/l)	349 (328–388) *n* = 9	340 (276–364) *n* = 14	329 (278–415) *n* = 25	323 (283–368)^*^n = 19	323 (274–353)^*^n = 16	328 (283–370) *n* = 13	335 (309–353) *n* = 7	**0.033**
Reticulocytes (10–110 10^9^l)	42 (20–203) *n* = 8	120 (9–328) *n* = 14	148 (7–318) *n* = 25	149 (13–495) *n* = 18	227 (11–451) *n* = 15	148 (58–437) *n* = 13	75 (17–242) *n* = 7	**0.046**
WBC (5.05–16.76 10^9^/l)	15.3 (7.4–58.6) *n* = 9	21.3 (5.9–50.8) *n* = 14	17.3 (7.8–65.5) *n* = 25	15.3 (7.9–48.9) *n* = 19	15.2 (4.0–33.4) *n* = 16	10.4 (6.4–17.4)^x, #^ *n* = 13	12.6 (6.4–19.3) *n* = 7	**0.007**
PLT (148– 484 10^9^/l)	339 (100–637) *n* = 9	395 (73–925) *n* = 14	436 (50–857) *n* = 25	370 (104–1,193) *n* = 19	446 (292–1,335) *n* = 16	545 (254–1,946) n = 13	553 (229–853) *n* = 7	0.141
Bilirubin (0–5.26 μmol/l)			4.1 (1.0–84.2) *n* = 20					
TP (55.5– 77.6 g/l)			55.2 (31.6–64.1) *n* = 18					
Alb (31.3– 43 g/l)			30.4 (18.0–46.1) *n* = 20					
HBs (%)			26.5 (2.4–52.2) *n* = 25	17.4 (1.7–42.1) *n* = 16	11.4 (2.0–32.0) *n* = 14	6.0 (0.0–11.7)^#^ *n* = 13	1.3 (0.0–6.0)^#, t, y^ *n* = 7	**< 0.001**
Eccentrocytes (%)			2.6 (0.0–15.8) *n* = 21	1.2 (0.0–7.2) *n* = 13	0.2 (0.0–7.8)^#^ *n* = 12	0.0 (0.0–0.8)^#^ *n* = 9	0.0 (0.0–0.0)^#, t^ *n* = 5	**< 0.001**

Not all parameters were available for each animal at every time point.

HB, Heinz body; HCT, hematocrit; HGB, hemoglobin; MCH, mean corpuscular hemoglobin; MCHC, mean corpuscular hemoglobin concentration; MCV, mean corpuscular volume; n: number of patients; PLT, platelets; RBC, red blood cells; WBC, white blood cells; checkup −2: day −10 to −4; checkup −1: day −3 to −1; checkup 0: day of HB diagnosis; checkup 1: day 1–3; checkup 2: day 4–6; checkup 3: day 7–13; checkup 4: day 14–20;^*^: different to checkup −2; ^x^: different to checkup −1;^#^: different to day 0, ^t^: different to checkup 1; ^y^: different to checkup 2; statistical analysis: Kruskal Wallis test with *post hoc* Dunn's multiple comparison.

On day 0, the median HCT was 23.8% (14.2–34.9%), with a median of 26.5% (2.4–52.2%) HBs and 2.6% (0.0–15.8%) eccentrocytes (Table 2). Data for eccentrocytes were unavailable for four dogs. HBs decreased from day 0 to following checkups and were significantly lower at checkup 3 and 4 compared to day 0 and decreased at checkup 4 compared to checkup 1 and 2. Eccentrocytes decreased at checkup 2, 3 and 4 compared to day 0 and at checkup 4 compared to checkup 1 (Table 2). The time course of changes in HCT an HBs is visualized in Figure 2. The course of eccentrocytes and reticulocytes are available in the Supplementary Figures 1, 2.

**Figure 2:**
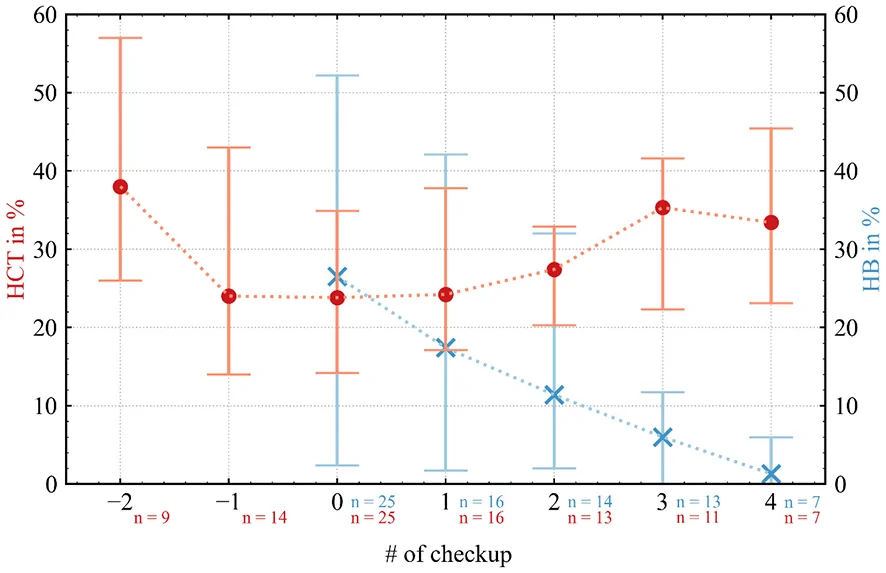
Course of median values of hematocrit and Heinz bodies from day −2 to checkup 4 including range of test samples (matplotlib in python), x-axis: #, number of checkup; y-axis: hematocrit and Heinz bodies in %; n, number of study participants; median, minimum and maximum values.

Data from day 0 and checkup 1 were available for all dogs at these time points. Reticulocyte count increased (*p* = 0.008), HBs (*p* = 0.014) and eccentrocytes (*p* = 0.004) decreased significantly (Table 3). Analyzing all dogs with complete data sets from day 0 to checkup 4, RBCs and HGB increased from checkup 2 to checkup 4 (*p* = 0.002; *p* = 0.013), MCV decreased from checkup 2 to checkup 4 (*p*= 0.016) and HBs decreased form checkup 1 to checkup 4 (*p* = 0.006; Table 4). Detailed data for additional time points are provided in the Supplementary Tables 3, 4.

**Table 3:** Comparison of complete blood count, Heinz bodies and eccentrocytes in patients with metamizole-associated Heinz body anemia between checkup 0 and 1.

Value	*n*	Checkup 0	Checkup 1	*p*-value
RBC (5.65–8.87 10^12^/l)	16	3.6 (2.0–5.0)	3.2 (2.4–5.4)	0.289
HCT (37.3–61.7 l/l)	16	0.25 (0.16–0.35)	0.24 (0.17–0.38)	0.660
HGB (13.1–20.5 mmol/l)	16	5.2 (3.1–7.7)	4.9 (3.3–8.1)	0.660
MCV (61.6–73.5 fl)	19	72.0 (61.1–87.1)	72.5 (62.8–88.2)	0.099
MCH (21.2–25.9 pg)	19	23.2 (21.5–33.8)	24.1 (21.3–25.6)	0.081
MCHC (32–37.9 g/l)	19	333 (280–415)	323 (283–368)	0.137
Reticulocytes (10–110 10^9^l)	18	138 (21–317)	149 (13–494)	**0.008**
WBC (5.05–16.76 10^9^/l)	19	18,7 (7.8–65.5)	15.3 (7.9–48.9)	0.365
PLT (148–484 10^9^/l)	19	436 (50–857)	370 (104–1,193)	0.481
HBs (%)	16	27.5 (6.6–52.2)	17.4 (1.7–42.1)	**0.014**
Eccentrocytes (%)	12	3.6 (0.0–15.8)	0.9 (0.0–7.2)	**0.004**

Data were available for the same study participants at checkup 0 and 1.

HB, Heinz body; HCT, hematocrit; HGB, hemoglobin; MCH, mean corpuscular hemoglobin; MCHC, mean corpuscular hemoglobin concentration; MCV, mean corpuscular volume; n: number of patients; PLT, platelets; RBC, red blood cells; WBC, white blood cells; checkup 0: day of HB diagnosis; checkup 1: day 1–3; statistical analysis: Friedman test with *post hoc* Dunn's multiple comparison test.

**Table 4:** Comparison of complete blood count, Heinz bodies and eccentrocytes of patients with metamizole-associated Heinz body anemia between checkup 0–4.

Value	*n*	Checkup 0	Checkup 1	Checkup 2	Checkup 3	Checkup 4	*p*-value
RBC (5.65– 8.87 10^12^/l)	6	4.0 (3.2–5.0)	4.1 (2.5–5.4)	4.0 (2.4–4.6)	4.6 (3.4–5.7)	4.8 (3.5–6.2)^y^	**0.002**
HCT (37.3– 61.7 l/l)	6	0.28 (0.24–0.35)	0.28 (0.20–0.38)	0.29 (0.20–0.33)	0.33 (0.22–0.42)	0.34 (0.23–0.45)	**0.024**
HGB (13.1– 20.5 mmol/l)	6	6.0 (4.6–7.7)	5.7 (3.7–8.1)	5.8 (4.0–7.0)	7.3 (4.9–8.7)	6.8 (5.0–9.4)^y^	**0.013**
MCV (61.6– 73.5 fl)	6	67.9 (63.3–75.6)	69.8 (66.0–81.9)	72.2 (68.8–97.1)	71.7 (65.0–89.7)	70.0 (61.2–75.1)^y^	**0.016**
MCH (21.2– 25.9 pg)	6	23.1 (21.9–24.8)	23.8 (21.3–24.8)	24.3 (21.6–26.7)	24.1 (21.3–25.5)	23.1 (20.9–26.4)	0.233
MCHC (32– 37.9 g/l)	6	343 (303–355)	330 (293–350)	323 (274–353)^#^	331 (283–354)	339 (309–353)	0.133
Reticulocytes (10–110 10^9^l)	5	61 (21–245)	100 (13–313)	123 (11–409)	93 (58–321)	64 (17–242)	0.326
WBC (5.05– 16.76 10^9^/l)	6	17.8 (8.8–19.4)	12.5 (9.4–25.8)	10.2 (4.0–18.2)	10.2 (6.6–12.6)	13.4 (6.4–19.3)	0.070
PLT (148– 484 10^9^/l)	6	534 (223–674)	526 (335–879)	443 (366–914)	530 (348–662)	573 (229–853)	0.900
HBs (%)	5	27.1 (9.7–45.0)	27.7 (6.9–35.5)	14.3 (3.5–32.0)	6.1 (0.0–11.7)	1.3 (0.0–6.0)^#, t^	**0.006**
Eccentrocytes (%)	2	2.6–15.8	0.6–7.2	0.4–7.8	0.0–0.2	0.0–0.0	**0.042**

Data were available for the same study participants at checkup 0–4.

HB, Heinz body; HCT, hematocrit; HGB, hemoglobin; MCH, mean corpuscular hemoglobin; MCHC, mean corpuscular hemoglobin concentration; MCV, mean corpuscular volume; n: number of patients; PLT, platelets; RBC, red blood cells; WBC, white blood cells; checkup −2: day −10 to −4; checkup −1: day −3 to −1; checkup 0: day of HB diagnosis; checkup 1: day 1–3; checkup 2: day 4–6; checkup 3: day 7–13; checkup 4: day 14–20; ^#^: different to day 0; ^t^: different to checkup 1; ^y^: different to checkup 2; statistical analysis: Friedman test with *post hoc* Dunn's multiple comparison test.

Decreased total protein and albumin, which may indicate gastrointestinal bleeding were observed in 10/25 dogs (Table 2). Mean HCT of dogs with suspected gastrointestinal bleeding (26.2 ± 4.2%) was not different from the HCT of dogs with without suspected gastrointestinal bleeding (21.8 ± 6.6%; *p* = 0.0642; Figure 3).

**Figure 3:**
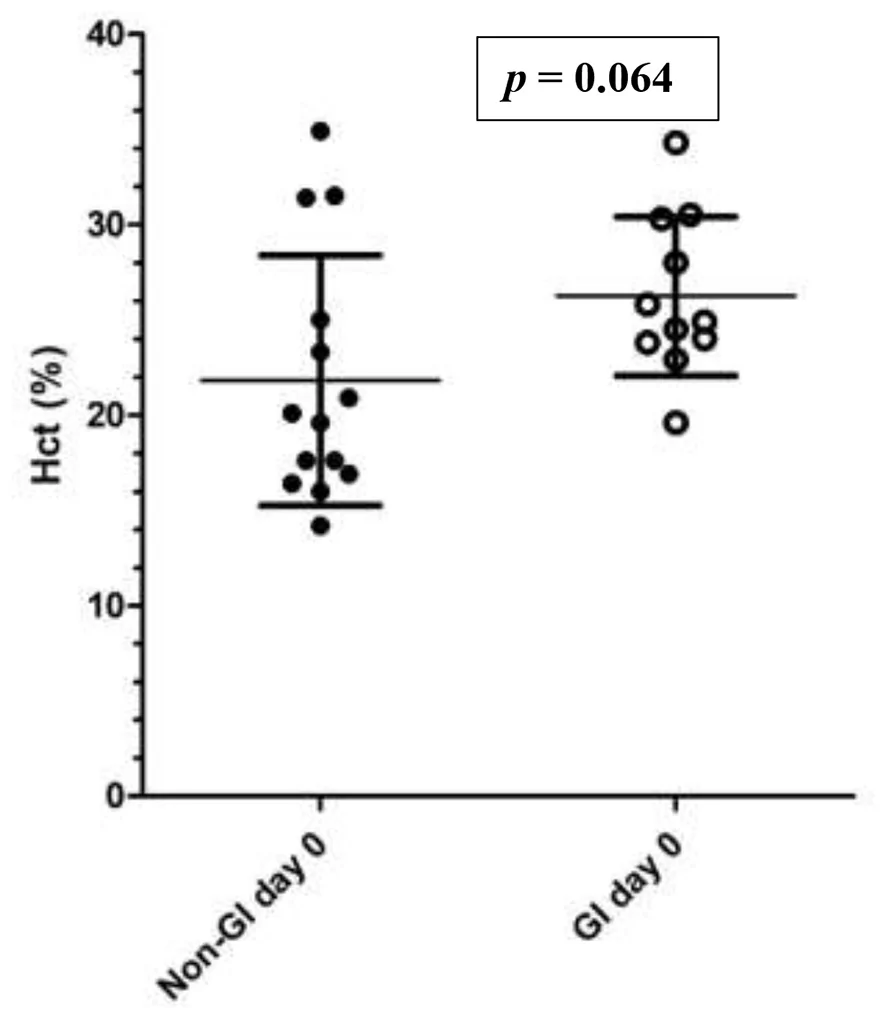
Hematocrit (HCT) on day 0 of dogs with metamizole-associated Heinz body anemia without (Non-GI) and with (GI) suspected gastrointestinal hemorrhage.

No correlation was found on day 0 between the amount of HBs and eccentrocytes, HCT, daily metamizole dose, or cumulative metamizole dose (Table 5). A moderate to strong correlation was identified between the amount of HBs and reticulocytes (*p* = 0.007; *r* = 0.630). Eccentrocyte count did not correlate with any of the parameters.

**Table 5:** Spearman correlation analysis between Heinz body and eccentrocyte counts and selected parameters at day 0 in 25 dogs receiving metamizole.

Parameter	Correlation with HBs		Correlation with eccentrocytes	
	***r***	***p*** **-value**	***r***	***p*** **-value**
HCT (%)	−0.189	0.415	−0.220	0.339
Reticulocytes (x 10^9^/l)	**0.630**	**0.007**	0.288	0.206
HBs (%)			0.200	0.385
Eccentrocytes (%)	0.200	0.385		
Cumulative metamizole dose (mg/kg)	0.241	0.293	−0.127	0.583
Daily metamizole dose (mg/kg/d)	−0.263	0.249	−0.027	0.906

HB, Heinz body; HCT, hematocrit; *r* = Spearman correlation coefficient; *p-*values < 0.05 were considered statistically significant.

All patients had at least one concurrent disease. Concurrent diseases by etiology and the medications used during the week before the onset of HB anemia were listed in Table 6 and Supplementary Table 5. Diseases included enteropathies, neoplasia, inflammatory diseases, nephropathies, and cardiopathies. Other medications administered included the antibiotics amoxicillin–clavulanic acid, marbofloxacin, metronidazole, and doxycycline.

**Table 6:** Underlying diseases by etiology and administered medications of 25 dogs with metamizole induced Heinz body anemia.

Patient	Disease by etiology	Other drugs
1	Inflammatory, toxic	Analgesia, antibiotics, antinausea, antiparasitics, blood products, gi-protection, probiotics
2	Infectious, inflammatory	Antibiotics, analgesia, antinausea, human albumine, gi-protection
3	Inflammatory	Immunosuppressants, gi-protection
4	Neoplastic	Analgesia, anesthetics, antibiotics, antinausea, blood products, probiotics
5	Idiopathic, inflammatory, neoplastic, infectious	Antibiotics, antiepileptics, gi-protection
6	Degenerative, metabolic	Analgesia, anesthetics, antibiotics, immunosuppressants
7	Degenerative, infectious, inflammatory	Analgesia, antibiotics, antinausea, gi-protection, heart medication, probiotics/prebiotics
8	Infectious, inflammatory, metabolic	Anesthetics, antiparasitics
9	Infectious, metabolic, inflammatory	Analgesia, anesthetics, antibiotics, antinausea, antiparasitics
10	Allergic, neoplastic, inflammatory	Analgesia, antibiotics, antinausea
11	Degenerative	Antinausea, gi-protection, heart medication
12	Idiopathic, obstructive	Antiepileptics, antinausea, blood products
13	Allergic, degenerative, metabolic, inflammatory	Analgesia, antinausea, antiparasitics, gi-protection, probiotics
14	Degenerative, neoplastic, inflammatory	Analgesia
15	Metabolic, infectious, degenerative	Antibiotics
16	Allergic, inflammatory	Antibiotics, antinausea, blood products, gi-protection, immunosuppressants
17	Neoplastic, degenerative	Analgesia, antibiotics
18	Neoplastic, degenerative, infectious, inflammatory	Antibiotics, heart medication
19	Inflammatory, infectious	Anesthetics, antibiotics, antinausea, probiotics
20	Allergic, inflammatory/immunmediated	Analgesia, anesthetics, antiparasitics, antinausea, blood products, immunosuppressants, probiotics
21	Degenerative, neoplastic	Antinausea, gi-protection
22	Neoplastic	Gi-protection, others
23	Inflammatory, neoplastic, infectious	Analgesia, anesthetics, antibiotics, antinausea, gi-protection, others
24	Degenerative, metabolic	Analgesia, gi-protection, others
25	Metabolic	Antibiotics, antinausea, gi-protection, others

GI, gastrointestinal.

## 4 Discussion

This prospective study evaluated dogs with metamizole-associated HB anemia, which was detected after a median of 10 days of treatment at recommended dosages and a median cumulative dose of 720 mg/kg. At detection of HB anemia, the median HCT was 24% with signs of regeneration in 15/25 dogs. Four patients showed clinical signs of anemia and received blood transfusions, however, all four patients survived. Oxidative damage was present, primarily in the form of HBs, with median percentage of 26.5% and occurred in the recommended dosage range of metamizole. This is an important difference from the experimental study by Kramer, in which significant oxidative damage was only found above the recommended dosage range. Metamizole was administered in six healthy dogs over 4 weeks at doses up to 450 mg/kg. Only IV administration of 450 mg/kg resulted in a significant increase in HBs, reticulocytes, while HGB, HCT, erythrocyte and white blood cell numbers decreased. After 4 weeks an elevation of blood urea nitrogen, bilirubin and alkaline phosphatase was observed (27). In a chronic toxicity study, six dogs received an oral dose up to 600 mg/kg/day metamizole over 26 weeks. A significant amount of HBs was identified at doses of 300 and 600 mg/kg. However, the study does not indicate the time at which HBs were present, nor does it specify the exact numbers or percentage of affected erythrocytes, limiting direct quantitative comparison with the present study (27). In another prospective study of 22 dogs receiving metamizole within the recommended dosage range (120 mg/kg/d) for at least 10 days, oxidative damage occurred only in form of eccentrocytes. A substantial increase in reticulocytes, as well as a decrease in hemoglobin was observed. In the metamizole group, 40.9% of dogs were anemic. Increased eccentrocyte counts were detected in 11/22 dogs, of which 5/11 also showed anemia. In the control groups that received carprofen or meloxicam, no eccentrocytes were detected in any of the dogs. However, the presence of HBs was not detected in any groups overall (24). Dogs in the present study were older compared to the study of Lieser et al. (24) (median age 9 vs. 6 years) and all patients showed concurrent disease, whereas comorbidities were excluded in the former study.

Due to the concurrent disease the question arises whether oxidative stress and the resulting damage in the erythrocytes might be multifactorial and not only due to metamizole. Erythrocytes have several protective mechanisms against oxidative stress, including glutathione, a tripeptide composed of the amino acids cysteine, glycine and glutamic acid. Reduced glutathione possesses a reactive sulfhydryl group, which can be oxidized (28, 29). Animals are continuously exposed to low levels of reactive oxygen (ROS) and nitrogen species (RNS). They are by-products of cellular metabolism, especially in the mitochondrial respiratory chain. Oxidative stress arises when the balance between pro-oxidant and antioxidant systems is disrupted (30). Oxidative stress in chronic disease is considered a multifactorial process. In critically ill patients, including those with systemic inflammatory conditions, ROS generation can increase markedly while antioxidant defenses may become depleted, thereby increasing susceptibility to oxidative damage (31). For example, patients with chronic kidney disease show evidence of altered oxidative stress markers and may exhibit compensatory antioxidant responses (32, 33). These findings suggest that concurrent disease may increase susceptibility to oxidative stress, while additional stressors may further challenge antioxidant capacity and contribute to oxidative erythrocyte damage. Further studies are needed to clarify the specific contribution of these mechanisms.

Furthermore, metamizole is rapidly hydrolyzed to its active metabolite and undergoes hepatic metabolism. In human *in vitro* studies, its metabolite 4-methylaminoantipyrine has been shown to enhance hemin toxicity in HL-60 cells by reducing cellular ATP levels via inhibition of glycolysis, thereby increasing cellular stress (22, 34, 35). These findings therefore provide a possible mechanism by which metamizole or its metabolites may contribute to cellular stress; however, the relevance of this mechanism to erythrocyte oxidative damage in dogs remains uncertain. Thus, patients in this study may have had increased oxidative stress due to concurrent disease, which may have been further exacerbated by metamizole. This could promote oxidative damage to erythrocytes, resulting in the formation of HBs and eccentrocytes without necessarily causing HB anemia in general. Further studies are needed to clarify the contribution of these individual factors.

Furthermore reduced antioxidant defense mechanisms in older patients (36, 37) may partly explain the predominance of older dogs in the present study. However, the occurence in a 2-year old dog indicates that age alone is not sufficient to explain these findings. Increasing age is also associated with a higher likelihood of underlying disease. Given the small sample size no firm conclusions can be drawn. HB-associated anemia appears to be primarily associated with metamizole exposure, although the contribution of concurrent disease and other patient-related factors to individual susceptibility cannot be excluded.

After discontinuing metamizole therapy and initiation of antioxidant therapy, hematocrit increased and the number of HBs and eccentrocytes decreased in the dogs of the current study. However, the specific contribution of antioxidant therapy to these changes cannot be determined, as there was no untreated control group. Only one dog received NAC, whereas the remaining dogs received SAMe. Given the different antioxidant agents and the very limited number of dogs receiving NAC, the potential effects of the two treatments on recovery could not be meaningfully compared. Webb et al. (38) demonstrated a protective effect of S-adenosylmethionine (SAMe) against acetaminophen-induced oxidative injury in cats. The decline in HBs after discontinuation of metamizole, together with initiating antioxidant therapy, supports the role of metamizole in oxidative erythrocyte damage.

Surprisingly, no correlation was found between HBs and eccentrocytes, although both represent manifestations of oxidative damage. HBs arise from oxidative damage to hemoglobin, whereas eccentrocytes are associated with oxidative injury to the erythrocyte membrane and cytoskeletal structures (39). In the present study, eccentrocytes were detected at low percentages in all but one dog. Values >10% were found in three dogs only. Eccentrocyte concentration decreased over time, similar to HBs. Median eccentrocyte concentration (2.6%) was comparable to those reported by Lieser et al., where the median eccentrocyte count in the metamizole group was 14.5 per 500 erythrocytes (corresponding to 2.9%) (24). Eccentrocytes have been observed in association with and independent of HBs (39, 40), and their underlying pathophysiological mechanisms remain incompletely understood. Certain diseases, such as diabetes mellitus or lymphoma, which can lead to the formation of HBs in cats, were described in dogs to induce eccentrocytes only (39, 41). However, the studies in cats do not specify whether eccentrocytes were also examined. The appearance of HBs and eccentrocytes likely depends on species-specific factors as well as the cause of the oxidative insult. Further investigation is warranted to explain this phenomenon.

No correlation was found between the number of HBs, eccentrocytes and HCT. It is possible that in certain patients HBs were already present for several days before diagnosis and the peak of HB formation had already occurred. This may explain the moderate-to-strong correlation, according to Cohen-type guidelines, between HBs and reticulocytes, as reticulocytosis typically develops with 3–5 days delay. However, this correlation may also be explained by the severity of concurrent disease or hemolysis, which could contribute to both increased HB formation and reticulocytosis, rather than necessarily reflecting a direct mechanistic relationship between the two parameters.

Some dogs additionally showed signs of gastrointestinal bleeding. However, hypoalbuminemia is non-specific and may also result from inflammation, protein-losing enteropathy or hepatic disease. Because many dogs in the present study had concurrent disease, these findings should be interpreted with caution. No difference in HCT was found between dogs with and without gastrointestinal bleeding on day 0. In addition, dogs without gastrointestinal bleeding had lower HCT levels. Therefore, gastrointestinal bleeding is unlikely to explain the observed findings.

Furthermore, no correlation was found between the percentage of HBs or eccentrocyte and daily or cumulative metamizole dose. Within the recommended dosage range, no clear dose-dependent relationship was observed; however, no control group receiving higher doses was available for comparison. As previously stated, HBs may have been present for several days prior to diagnosis, potentially obscuring a dose-response relationship. It is also possible that individual variability in susceptibility contributes to the observed findings.

As described above, 4 dogs received a blood transfusion during the study period. Therefore, HCT, RBC, and HGB were excluded from the following analyses, as these parameters can be substantially affected by transfusion. Although transfusion may also influence other variables, including HBs and reticulocyte counts, its effect on these parameters was considered limited, particularly for the assessment of HB regression over time.

Severe hematological adverse drug reactions are generally considered idiosyncratic and not classically dose-dependent (42–44), although this has not been reported in dogs to date. In the present study, two dogs (5, 24) repeatedly exhibited anemia following additional metamizole administration. Unfortunately, no further details are available to investigate this phenomenon. It is conceivable that an idiosyncratic cause is involved. Additionally, no correlation was found between elapsed time, from the start of metamizole therapy to the HB diagnosis and the of HBs on day 0. However, the study population is too small to draw any general conclusions about this matter. As discussed above, the absence of a control group means that no definitive conclusions can be drawn regarding a potential dose-independent effect of metamizole.

Given the known causes of HB anemia, the dogs included in our study were examined for other potential causes. Patient No. 4 was diagnosed with lymphoma. To the authors knowledge, T-cell lymphoma can cause oxidative damage in dogs in the form of eccentrocytes, but not in the form of HBs (39). It can be assumed that the neoplasia caused further oxidative stress in this patient, but with a total of maximum 0.6% eccentrocytes, this does not appear to have had a significant impact. Another more precisely defined tumor was an insulinoma in patient No. 10. To the authors knowledge, no oxidative damage has been described in this context to date. In cats, HB anemia has been described as a consequence of repeated administration of propofol. To the authors knowledge, there is only one study that has shown HBs in dogs that received propofol DTI over a period of several days (16). Therefore, a connection between regular anesthesia lasting a few hours, as in patients 4, 6, 8, 9, 19, 20, 23 and HB anemia seems very unlikely. One dog in this study received zinc as a dietary supplement at a dose of approximately 0.05 mg/kg/day, which does not exceed the recommended maximum daily dose of zinc (45). It is unlikely that this dose of zinc has any effect on HB anemia, as significantly larger amounts of zinc were ingested in the cases of zinc intoxication described (6, 46). To the best of the authors' knowledge the other medications administered (Supplementary Table 5) have not been reported to cause HB formation or HB anemia in dogs.

Dogs with low amounts of HBs counts ( ≤ 5%) were excluded from the study as this is considered clinical irrelevant (25). As discussed above, anemia maybe multifactorial and cannot be reliably attributed to low HBs alone.

Metamizole-associated HB anemia is a reversible side effect as the hematocrit increased and the number of HBs and eccentrocytes decreased after discontinuing metamizole therapy and starting antioxidant therapy. In the author's clinic, 25 cases were identified during the 4-year collection period. As metamizole is a commonly used medication in the author's clinic and in the surrounding area, a substantially larger number of dogs likely received this treatment during the study period. However, the exact number of exposed patients was not determined, and therefore these findings should be interpreted with caution and considered only as preliminary indications. Although this side effect appears to be rare, no definitive conclusions regarding its true frequency can be drawn due to the lack of incidence data.

The prognosis for HB anemia is generally favorable, but is significantly influenced by the presence and course of additional diseases and the severity of the anemia. As previously described, the deaths of the four patients occurred predominantly due to severe complications from concurrent disease and not exclusively due to HB anemia. The death of the patient with mitral valve endocardiosis was not HB anemia associated at all. It should be noted that HB anemia can become so severe that patients require blood transfusions. This was the case for four patients in the present study, however, all four dogs survived.

Given the heterogeneous reasons for missing checkups, the missing data could not be reliably classified as missing at random and selection bias cannot be excluded. Some of these reasons may have been related to the clinical course, whereas others were likely unrelated to the hematological outcome. Because treatment settings were influenced by the severity of concurrent disease, a limitation of this study is that management was not standardized across all dogs. Furthermore, checkup assessments were performed within predefined time ranges rather than on fixed calendar days resulting in variable sampling times, and complete checkup data were not available for all dogs. Because the number of available dogs varied between individual checkup time points, the Kruskal–Wallis test was used for the analysis presented in Table 2. However, this approach does not consider repeated measurements from the same dogs across time points and therefore represents a limitation of the analysis. Furthermore, the analyses were limited to univariable Spearman correlation analyses. Therefore, potential confounding factors, including age, duration of metamizole treatment, cumulative dose, hospitalization, blood transfusion, and antioxidant therapy, could not be accounted for simultaneously. While the small sample size prevented reliable multivariable modeling, the potential impact of these factors on the observed associations should be considered when interpreting the results. Consequently, fewer dogs could be included in these analyses. In addition, the statistical analyses were performed using GraphPad Prism version 5. Although this software was sufficient for the statistical methods applied in this study, newer versions and alternative statistical software packages may offer additional analytical capabilities. Furthermore, the inclusion criteria of >5% HBs combined with the presence of anemia resulted in the selection of clinically apparent cases. Consequently, subclinical cases and dogs with only mild oxidative damage have been excluded.

## 5 Conclusion

This study described HB anemia as a potentially reversible and rare side effect of metamizole with resolution observed after discontinuation of the drug and initiation of antioxidant therapy. Dogs with concurrent disease represented all identified cases in this study, suggesting that these patients may represent a population at increased risk for this adverse effect; however, controlled studies are required to determine whether concurrent disease independently increases susceptibility. The use of metamizole over a period of several days (minimum 3 days) in these patients should be carefully considered and accompanied by regular hematology checkups.

## Data Availability

The original contributions presented in the study are included in the article/Supplementary material, further inquiries can be directed to the corresponding author.

## Supplementary material

Supplementary Figure 1 Course of median values of Reticulocytes from day −2 to checkup 4 including range of test samples (matplotlib in python), x-axis: timepoint checkup; y-axis: reticulocytes in 10^9^l; n: number of study participants; median, minimun and maximum values.

Supplementary Figure 2 Course of median values of eccentrocytes from day 0 to checkup 4 including range of test samples (matplotlib in python), x-axis: timepoint checkup; y-axis: eccentrocytes in %; n, number of study participants; median, minimun and maximum values.
