# Supplementary material for: Hematologic follow-up of metamizole-associated Heinz body anemia in dogs
Source: Front Vet Sci. 2026 Sep 28;13:1913770. doi: 10.3389/fvets.2026.1913770 (PMC13656853; doi:10.3389/fvets.2026.1913770)
Supplement: Supplementary file 1 [file Image_1.pdf]

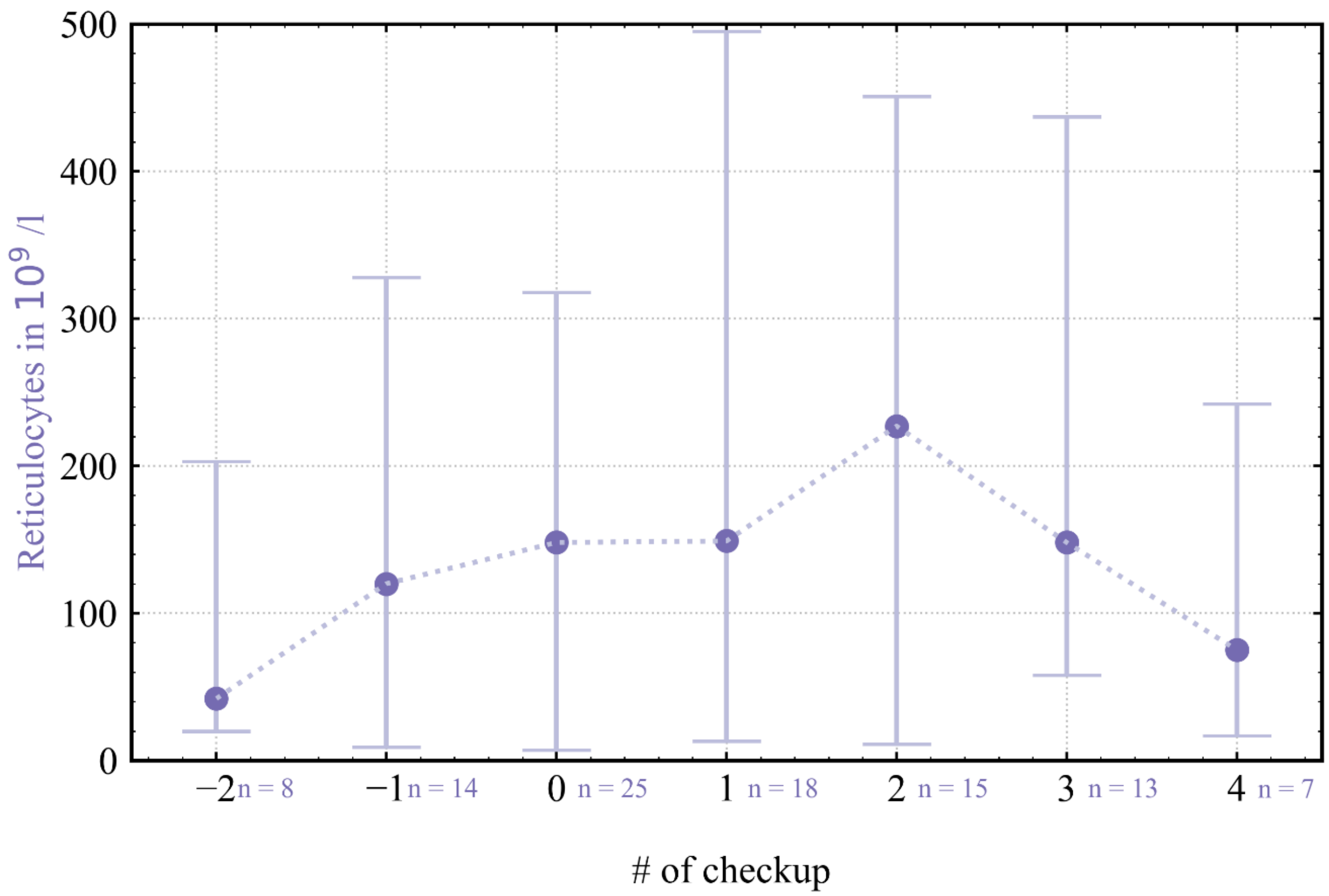

Figure 1: Course of median values of Reticulocytes from day -2 to checkup 4 including range of test samples (matplotlib in python), x-axis: timepoint checkup; y-axis: Reticulocytes in 10^9l; n: number of study participants; median, minimun and maximum values
