# Supplementary material for: Hematologic follow-up of metamizole-associated Heinz body anemia in dogs
Source: Front Vet Sci. 2026 Sep 28;13:1913770. doi: 10.3389/fvets.2026.1913770 (PMC13656853; doi:10.3389/fvets.2026.1913770)
Supplement: Supplementary file 2 [file Image_2.pdf]

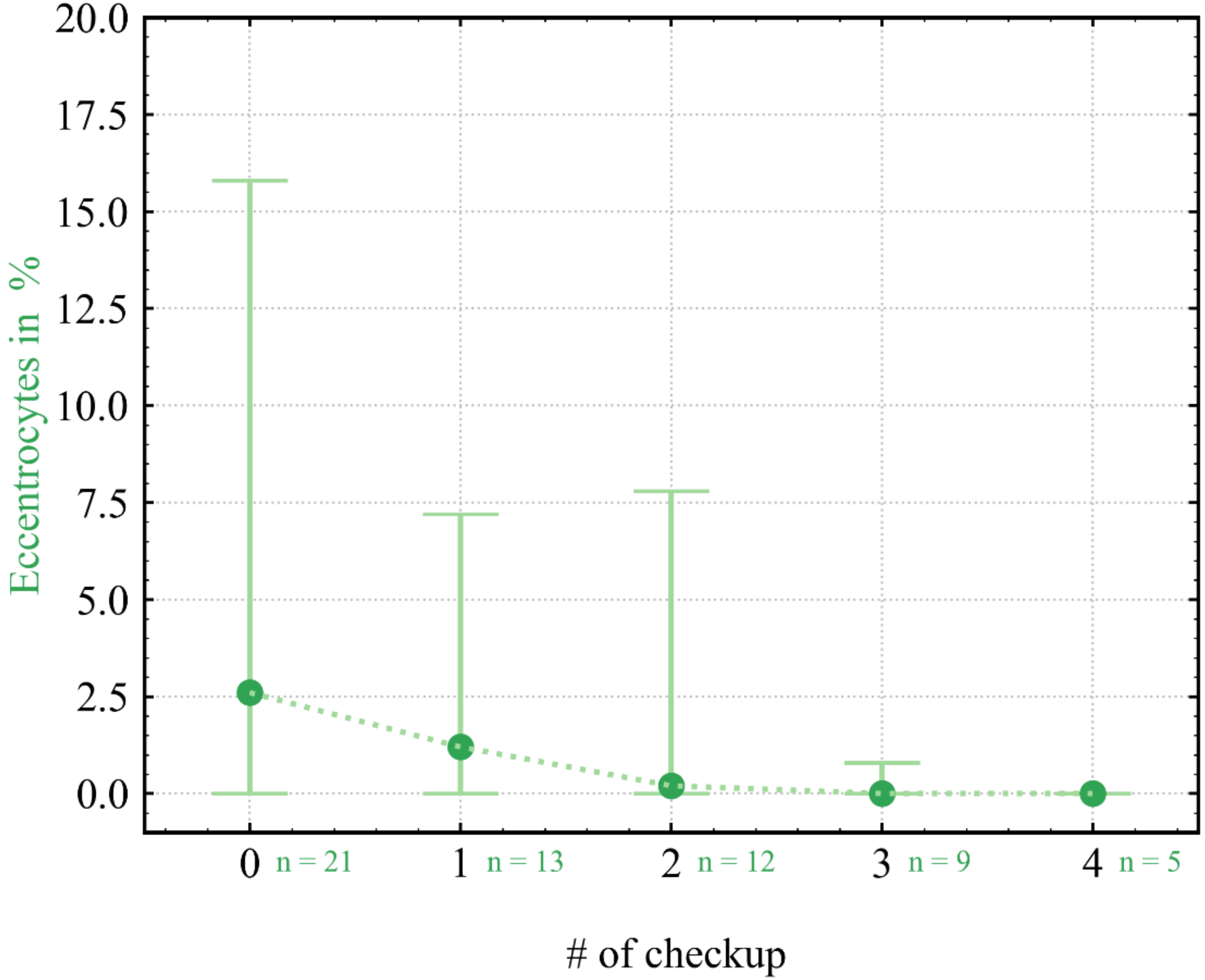

20.0
17.5
15.0
12.5
10.0
7.5
5.0
2.5
0.0
Eccentrocytes in %
0 n = 21
1 n = 13
2 n = 12
3 n = 9
4 n = 5
# of checkup

Figure 2: Course of median values of eccentrocytes from day 0 to checkup 4 including range of test samples (matplotlib in python), x-axis: timepoint checkup; y-axis: Eccentrocytes in %; n: number of study participants; median, minimun and maximum values
