# Supplementary material for: Hematologic follow-up of metamizole-associated Heinz body anemia in dogs
Source: Front Vet Sci. 2026 Sep 28;13:1913770. doi: 10.3389/fvets.2026.1913770 (PMC13656853; doi:10.3389/fvets.2026.1913770)
Supplement: Supplementary file 3 [file Table_1.docx]

**Table 1: dosage/application metamizole (mg/kg), duration (days), hospitalization (days) of 25 dogs with Heinz body anemia after receiving metamizole:**

| **Parameter** | **Unit** | **Samples n** | **Median** | **Range** |
| --- | --- | --- | --- | --- |
| **Dosage/application metamizole** | mg/kg | 31 | 35 | 20–60 |
| **Duration** | Days | 23 | 8 | 1–51 |
| **Hospitalization** | Days | 21 | 6 | 1–17 |
