# Supplementary material for: Hematologic follow-up of metamizole-associated Heinz body anemia in dogs
Source: Front Vet Sci. 2026 Sep 28;13:1913770. doi: 10.3389/fvets.2026.1913770 (PMC13656853; doi:10.3389/fvets.2026.1913770)
Supplement: Supplementary file 4 [file Table_2.docx]

**Table S2:** **Complete blood count and selected laboratory parameters of study participants with Heinz body anemia before, at, and after detection of the Heinz bodies. Not all parameters were available for each animal at every time point:**

| Value | Checkup -2 | Checkup -1 | Checkup 0 | Checkup 1 | Checkup 2 | Checkup 3 | Checkup 4 |
| --- | --- | --- | --- | --- | --- | --- | --- |
| n | 9 | 14 | 25 | 16 | 13 | 11 | 7 |
| RBC ↓, ↔, ↑ | 2, 7, 0 | 13, 1, 0 | 25, 0, 0 | 16, 0, 0 | 13, 0, 0 | 10, 1, 0 | 6, 1, 0 |
| n | 9 | 14 | 25 | 16 | 13 | 11 | 7 |
| HCT ↓, ↔, ↑ | 4, 5, 0 | 13, 1, 0 | 25, 0, 0 | 15, 1, 0 | 13, 0, 0 | 10, 1, 0 | 6, 1, 0 |
| n | 9 | 14 | 25 | 16 | 13 | 11 | 7 |
| HGB ↓, ↔, ↑ | 4, 4, 1 | 13, 1, 0 | 25, 0, 0 | 16, 0, 0 | 13, 0, 0 | 10, 1, 0 | 6, 1, 0 |
| n | 9 | 14 | 25 | 19 | 16 | 13 | 7 |
| MCV ↓, ↔, ↑ | 3, 6, 0 | 2, 9, 3 | 1, 15, 9 | 0 ,12, 7 | 0, 7, 9 | 0, 7, 6 | 1, 4, 2 |
| MCH ↓, ↔, ↑ | 2, 7, 0 | 1, 13, 0 | 1, 23, 1 | 0, 19, 0 | 0, 14, 2 | 0, 12, 1 | 1, 5, 1 |
| MCHC ↓, ↔, ↑ | 0, 8, 1 | 5, 9, 0 | 7 ,17, 1 | 8, 10, 0 | 7, 9, 0 | 4, 9, 0 | 1, 6, 0 |
| n | 8 | 14 | 25 | 18 | 15 | 13 | 7 |
| Reticulocytes ↔, ↑ | 7, 1 | 7, 7 | 10, 15 | 7, 11 | 5, 10 | 6, 7 | 5, 2 |
| n | 9 | 14 | 25 | 19 | 16 | 13 | 7 |
| WBC ↓, ↔, ↑ | 0, 6, 3 | 0, 5, 9 | 0, 9, 16 | 0, 10, 9 | 1, 9, 6 | 0, 12, 1 | 0, 6, 1 |
| n | 9 | 14 | 25 | 19 | 16 | 13 | 7 |
| PLT ↓, ↔, ↑ | 2, 5, 2 | 2, 8, 4 | 2, 13, 10 | 2, 9, 8 | 0, 9, 7 | 0, 4, 9 | 0, 1, 6 |
| n |  |  | 25 | 16 | 14 | 13 | 7 |
| HBs: ↔, ↑ |  |  | 0, 25 | 0, 16 | 0, 14 | 1, 12 | 3, 4 |
| n |  |  | 21 | 13 | 12 | 9 | 5 |
| Eccentrocytes ↔, ↑ |  |  | 1, 20 | 2, 11 | 6, 6 | 5, 4 | 5, 0 |
| n |  |  | 21 | 13 | 12 | 9 | 5 |
| Bilirubin ↔, ↑ |  |  | 13, 7 |  |  |  |  |
| n |  |  | 18 |  |  |  |  |
| Total protein ↓, ↔, ↑ |  |  | 10, 8, 0 |  |  |  |  |
| n |  |  | 20 |  |  |  |  |
| Albumin↓, ↔, ↑ |  |  | **10, 9, 1** |  |  |  |  |

HB: Heinz body; RBC: red blood cells; HCT: hematocrit; HGB: hemoglobin; MCV: mean corpuscular volume; MCH: mean corpuscular hemoglobin; MCHC: mean corpuscular hemoglobin concentration; WBC: white blood cells; PLT: platelets; n: number of patients;**↓**: under the reference range; ↔: in the reference range; ↑: over the reference range; checkup -2: day -10 to -4; checkup -1: day -3 to -1; checkup 0: day of HB diagnosis, checkup 1: day 1 to 3; checkup 2: day 4 to 6; checkup 3: day 7 to 13; checkup 4: day 14 to 20
