# Supplementary material for: Hematologic follow-up of metamizole-associated Heinz body anemia in dogs
Source: Front Vet Sci. 2026 Sep 28;13:1913770. doi: 10.3389/fvets.2026.1913770 (PMC13656853; doi:10.3389/fvets.2026.1913770)
Supplement: Supplementary file 5 [file Table_3.docx]

**Table S3:** **Comparison of Complete blood count, Heinz bodies and eccentrocytes in patients with Heinz body anemia between checkup 0, 1 and 2. Data were available for the same study participants at checkup 0, 1 and 2:**

| **Value** | **n** |  | **Checkup 0** | **Checkup 1** | **Checkup 2** | ***p*-value** |
| --- | --- | --- | --- | --- | --- | --- |
| **RBC (5.65–8.87 10^12^/l)** | 11 |  | 7.8 (2.0–5.0) | 7.3 (2.5–5.4) | 3.9 (2.4–4.6) | 0.732 |
| **HCT (37.3–61.7 l/l)** | 11 |  | 0.25 (0.18–0.35) | 0.25 (0.19–0.38) | 0.31 (0.20–0.33) | 0.403 |
| **HGB (13.1–20.5 mmol/l)** | 11 |  | 5.2 (3.1–7.7) | 5.0 (3.7–8.1) | 5.9 (4.0–7.0) | 0.351 |
| **MCV (61.6–73.5 fl)** | 14 |  | 69.4 (61.1–87.1) | 71.4 (62.8–88.2) | 76.4 (64.9–97.1) ^#^ | **0.002** |
| **MCH (21.2–25.9 pg)** | 14 |  | 23.3 (21.5–33.8) | 24.1 (21.3–25.6) | 24.4 (21.6–26.7) | 0.289 |
| **MCHC (32–37.9 g/l)** | 14 |  | 343 (284–415) | 325 (283–368) | 320 (274–353) ^#^ | **0.009** |
| **Reticulocytes (10–110 10^9^l)** | 12 |  | 104 (21–317) | 152 (13–494) | 235 (11–451) | 0.076 |
| **WBC (5.05–16.76 10^9^/l)** | 14 |  | 18.7 (7.8–65.5) | 18.7 (9.4–48.9) | 15.9 (4.0–33.4) ^#, t^ | **0.013** |
| **PLT (148–484 10^9^/l)** | 14 |  | 459 (142–857) | 435 (104–1193) | 446 (292–1335) | 0.257 |
| **HBs (%)** | 10 |  | 35.1 (6.6–52.2) | 27.7 (6.9–42.1) | 15.0 (2.0–32.0) | 0.067 |
| **Eccentrocytes (%)** | 6 |  | 5.6 (0.0–15.8) | 1.2 (0.0–7.2) | 0.0 (0.0–7.8) ^#^ | **0.008** |

HB: Heinz body; HCT: hematocrit; HGB: hemoglobin; MCH: mean corpuscular hemoglobin; MCHC: mean corpuscular hemoglobin concentration; MCV: mean corpuscular volume; ; n: number of patients; PLT: platelets; RBC: red blood cells; WBC: white blood cells; **↓**: under the reference range; ↔: in the reference range; ↑: over the reference range; checkup –2: day –10 to –4; checkup –1: day –3 to –1; checkup 0: day of HB diagnosis; checkup 1: day 1 to 3; checkup 2: day 4 to 6; checkup 3: day 7 to 13; checkup 4: day 14 to 20; ^#^:different to day 0; ^t^: different to checkup 1; statistical analysis: Friedmann test with post hoc Dunn´s multiple comparison test
