# Supplementary material for: Hematologic follow-up of metamizole-associated Heinz body anemia in dogs
Source: Front Vet Sci. 2026 Sep 28;13:1913770. doi: 10.3389/fvets.2026.1913770 (PMC13656853; doi:10.3389/fvets.2026.1913770)
Supplement: Supplementary file 6 [file Table_4.docx]

**Table S4:** **Comparison of complete blood count, Heinz bodies and eccentrocytes in patients with Heinz body anemia between checkup 0 - 3. Data were available for the same study participants at checkup 0 and 3:**

| **Value** | **n** | **Checkup 0** | **Checkup 1** | **Checkup 2** | **Checkup 3** | ***p*-value** |
| --- | --- | --- | --- | --- | --- | --- |
| **RBC (5.65–8.87 10^12^/l)** | 9 | 7.8 (2.9–5.0) | 3.5 (2.5–5.4) | 4.0 (2.4–4.6) | 4.7 (3.4–5.7) ^t, y^ | **0.009** |
| **HCT (37.3–61.7 l/l)** | 9 | 0.25 (0.20–0.35) | 0.25 (0.19–0.38) | 0.31 (0.20–0.33) | 0.36 (0.22–0.42) ^t^ | **0.017** |
| **HGB (13.1–20.5 mmol/l)** | 9 | 5.5 (4.2–7.7) | 5.1 (3.7–8.1) | 6.1 (4.0–7.0) | 7.3 (4.9–8.7) ^t, y^ | **0.01** |
| **MCV (61.6–73.5 fl)** | 10 | 68.8 (63.3–75.6) | 70.0 (66.0–81.9) | 73.5 (68.8–97.1) ^#^ | 74.0 (65.0–89.7) | **0.001** |
| **MCH (21.2–25.9 pg)** | 10 | 23.1 (21.9–24.8) | 23.8 (21.3–24.8) | 24.3 (21.6–26.7) | 24.1 (21.3–25.5) | 0.503 |
| **MCHC (32–37.9 g/l)** | 10 | 343 (303–355) | 330 (293–368) | 320 (274–353) ^#^ | 330 (283–354) | **0.012** |
| **Reticulocytes (10– 110 10^9^l)** | 8 | 104 (21–245) | 86 (13–460) | 224 (11–451) | 100 (58–321) | 0.070 |
| **WBC (5.05–16.76 10^9^/l)** | 10 | 17.8 (7.8–65.5) | 14.1 (9.4–48.9) | 16.1 (4.0–33.4) | 10.5 (6.6–12.6) ^t^ | **0.020** |
| **PLT (148–484 10^9^/l)** | 10 | 477 (142–674) | 435 (104–879) | 446 (292–914) | 551 (348–992) | 0.669 |
| **HBs (%)** | 8 | 27.1 (6.6–52.2) | 27.7 (6.9–42.1) | 15.0 (3.5–32.0) | 4.8 (0.0–11.7) ^#, t^ | **0.002** |
| **Eccentrocytes (%)** | 4 | 5.6 (0.0–15.8) | 0.9 (0.0–7.2) | 0.2 (0.0–7.8) | 0.0 (0.0–0.2) | **0.036** |

HB: Heinz body; HCT: hematocrit; HGB: hemoglobin; MCH: mean corpuscular hemolgobin; MCHC: mean corpuscular hemoglobin concentration; MCV: mean corpuscular volume; n: number of patients; PLT: platelets; RBC: red blood cells; WBC: white blood cells; **↓**: under the reference range; ↔: in the reference range; ↑: over the reference range; checkup –2: day –10 to –4; checkup –1: day –3 to –1; checkup 0: day of HB diagnosis; checkup 1: day 1 to 3; checkup 2: day 4 to 6; checkup 3: day 7 to 13; checkup 4: day 14 to 20;^#^: different to day 0; ^t^: different to checkup 1, ^y^: different to checkup 2; statistical analysis: Friedmann test with post hoc Dunn´s multiple comparison test
