# Supplementary material for: Hematologic follow-up of metamizole-associated Heinz body anemia in dogs
Source: Front Vet Sci. 2026 Sep 28;13:1913770. doi: 10.3389/fvets.2026.1913770 (PMC13656853; doi:10.3389/fvets.2026.1913770)
Supplement: Supplementary file 7 [file Table_5.docx]

**Table S5:** **Underlying diseases by specific diseases and specific administered medications of 25 dogs with metamizole associated Heinz body anemia. Patients that died during hospitalization are highlighted in black:**

| patient | conditions | analgesia | anesthethics | antibiotics | antiepileptics | antinausea | antiparasitics | blood products | gi protection | heartmedication | immunosuppressants | probiotics | others |
| --- | --- | --- | --- | --- | --- | --- | --- | --- | --- | --- | --- | --- | --- |
| 1 | AHDS | buprenor-phine, metha-done |  | amoxiclav |  | maropi-tant, MCP, ondan-setrone, prucalo-pride | imidacloprid, moxidectin | human albumin, blood transfusion (red blood cell concentra-te) | esome-prazole |  |  | lactic acid bacteria, bifido-bacteria |  |
| 2 | pyometra, pancrea-topathy, cholezys-topathy | buprenor-phine, FLK |  | amoxiclav |  | maropi-tant, MCP, ondan-setrone, prucalo-pride |  | human albumin | esome-prazole, sucrabest |  |  |  |  |
| 3 | IBD |  |  |  |  |  |  |  | omepra-zole, sucrabest |  | cyclosporine |  |  |
| 4 | ruptured spleen mass malignant lymphoma | metha-done | midazolam, propofol, isofloran | amoxiclav |  | maropi-tant |  | human albumin, 2 blood transfusion (full blood transfusion) |  |  |  | lactic acid bacte-ria, bifido-bacteria |  |
| 5 | epilepsy, chronic enteropa-thy, multiple masses at abdomen, anal sac abscess |  |  | marboflo-xacin | pheno-barbital |  |  |  | sucra-best, cimetidin |  |  |  |  |
| 6 | suspected AKI on CKD | metha-done, meloxi-cam | midazolam, propofol, ketamin, isofloran, lidocain, dopamine | amoxiclav |  |  |  |  |  |  | cyclopsporine |  |  |
| 7 | ME chief B2, aortic inssuficien-ce acute phase on chronic enteropa-thy, colangitis | buprenor-phine, xylocain gel |  | amoxiclav |  | maropi-tant, MCP |  |  | esome-prazole, surabest | pimoben-dan |  | lactic acid bacte-ria, bifido-bacte-ria, fibre |  |
| 8 | leishmania-sis, uveitis, nephrolith-iasis |  | medication unknown |  |  |  | allopuri-nol, telmisar-tan, |  |  |  | prednisolone, cyklopsorine, |  | ketorolac, dexpan-thenol, ferric sodium EDTA, lactotrans-ferrin, dorzola-mide, timolol, brinzolami-de |
| 9 | leishmania-sis, nephrolithi-asis with hydrone-phrosis and hydroureter, septical abdomen, pancreatitis | metha-done, fentanyl | midazolam, propofol, isofluran, dopamine | amoxiclav, marboflo-xacin |  | maropi-tant | allopuri-nol |  |  |  |  |  |  |
| 10 | allergies (environ-ment, food) spleen extirpation (splenic hyperpla-sia), insolinoma, sepsis post insulima operation; pancrea-titis | metha-done, buprenor-phine |  | metronidazole, amoxiclav, marboflo-xacin |  | maropi-tant, mirtaza-pine |  |  |  |  |  |  |  |
| 11 | ME chief c, lung edema |  |  |  |  | mirtaza-pine |  |  | esome-prazole | pimoben-dan, furosemide |  |  |  |
| 12 | epilepsy, foreign body removal (laparato-my) |  |  |  | pheno-barbital, levetir-acetam, pregaba-lin, CBD | maropi-tant |  | bloodtrans-fusion (full blood transfusion) |  |  |  |  |  |
| 13 | food allergy, AKI on CKD, suspected pancreatitis | buprenor-phine |  |  |  | maropi-tant, ondan-setrone, MCP | sarolaner |  | sucrose |  |  | lactic acid bacte-ria, bifido-bacte-ria |  |
| 14 | arthrosis, focal round mass thorax, mutiple partly necrotizing masses on the body | bedinvet-mab |  |  |  |  |  |  |  |  |  |  |  |
| 15 | bilateral SUB, recurrent cystitis, structural nephropa-thy |  |  | amoxiclav |  |  |  |  |  |  |  |  |  |
| 16 | suspected allergy, otitis interna/me-dia, hyperten-sion |  |  | amoxiclav, |  | maropi-tant, MCP |  | blood transfusion (red blood cell concentrate) | esomperazole, sucralfat |  | prednisolone |  | amlodipine |
| 17 | Intratho-racic mass, spondylo-sis, arthrosis, recurrent episodes of weakness | bedinvet-mab |  | doxycycline |  | meloxi-cam |  |  |  |  |  |  |  |
| 18 | mammary tumor, mitral and tricuspidal-insufficien-cy chief B2, pyometra, systemic inflamma-tion post OHE |  |  | amoxiclav |  |  |  |  |  | benazepril |  |  |  |
| 19 | acute on chronic enteropathy |  | dexmedetho-midine, ketamine, methadone, alfaxan, sevoflurane, fentanyle, glycopyrrolate | amoxiclav |  | maropi-tant, ondan-setrone, MCP |  |  |  |  |  | lactic acid bacte-ria, bifido-bacte-ria |  |
| 20 | chronic enteropa-thy, suspected PLE | bupenor-phine | ketamine, propofol, isofluran, glycopyrro-late. ephedrine, noradrena-line |  |  | maropi-tant | fenben-dazole | human albumine |  |  | chlorabucile, prednisolone | lactic acid bacte-ria, bifido-bacte-ria | clopidogrel |
| 21 | CNE, masses in liver, syncope, ME chief B1 |  |  |  |  | maropi-tant |  |  | sucrabest |  |  |  |  |
| 22 | cushing |  |  |  |  |  |  |  | sucralfat, omepra-zole |  |  |  | trilostane |
| 23 | prostate abscess, testicular tumor (sertoli-cell tumor), surgical site infection (post prostate marsupi-alization) | buprenor-phine, fentanyl | ketamin, metha-done, propofol, isofluran | amoxiclav, marboflo-xacine, |  | maropi-tant |  |  | sucrabest |  |  |  | chlorphe-namine |
| 24 | arthrosis, hypothy-erosis | gabapen-tine |  |  |  |  |  |  | elm bark |  |  |  | ursodesoxychol acid, propentohylline, vitamine B12, levothyro-xine |
| 25 | AKI post anesthe-sia |  |  | Amoxiclav, marboflo-xacine |  | maropi-tant, MCP, ondansetron |  |  | esome-prazole, sucrabest |  |  |  | calcium carbonate |
